# Biosimilar FSH preparations- are they identical twins or just siblings?

**DOI:** 10.1186/s12958-016-0167-8

**Published:** 2016-06-14

**Authors:** Raoul Orvieto, David B. Seifer

**Affiliations:** Department of Obstetrics and Gynecology, Infertility and IVF Unit, Chaim Sheba Medical Center (Tel Hashomer), Ramat Gan, 52621 Israel; Sackler Faculty of Medicine, Tel Aviv University, Tel Aviv, Israel; Department of Obstetrics and Gynecology, Division of Reproductive Endocrinology and Infertility, Dartmouth-Hitchcock Medical Center, Geisel School of Medicine at Dartmouth, Lebanon, NH USA

## Abstract

As patents expire on innovator products, there is increasing interest in developing biosimilar products globally. Biosimilars are not exact copies and are not considered generic versions of the reference product. They may differ in strength, purity and contain different composition of isoforms and/or various glycosylation profiles, with the consequent alterations in clinical efficacy or safety. Recently 2 new recombinant FSH preparations were introduced to clinical practice following randomized controlled, phase 3 clinical trials. Both, Bemfola and Ovaleap® were referred to the FSH innovator product Gonal-f™ (Follitropin alpha), and were found to yield an equivalent number of oocytes (primary end-point), following a long GnRH agonist suppressive protocol in “ideal” patients, i.e., young, normal responders. However, a closer look at these RCTs reveals a non-significant 4 % difference in clinical and ongoing pregnancy rates, in favor of Gonal f over the biosimilar products, accompanied by half the incidence of OHSS (2.9 vs 5.2 %, respectively). These studies were underpowered with reference to pregnancy rates, Thus, we believe that further comparative studies are needed in additional patient populations, e.g.,older,, poor responders, patients with repeated IVF failures and/or polycystic ovary syndrome, before the universal implementation of biosimilar products for clinical use. Biosimilars are actually a regulatory synonym, facilitating a fast track introduction of a FSH preparation to the COH armamentarium. We therefore recommend against interchanging or substituting innovator and biosimilar agents in clinical practice, and believe that the decision whether to use an innovator or a biosimilar product, should be reserved to the discretion of the treating physician. Furthermore, we believe the time has come that the measurement of the biological activity of FSH in humans should require other methods rather than the Steelman-Pohley assay, such as the determination of dose–response curves in defined populations of women with well-defined outcomes during COH in preparation for ART.

## Introduction

As patents expire on innovator products, there is increasing interest in developing biosimilar products globally. The FDA describes biosimilars as biologic products that are “highly similar to the reference product not with-standing minor differences in clinically inactive components and that there are no clinically meaningful differences between the biologic product and the reference product in terms of safety, purity, and potency of the product” [1]. This definition makes it clear that biosimilars are not identical molecules or “generics” for biologic agents. They may still differ in strength, purity and contain different composition of isoforms and/or various glycosylation profiles, with consequent alterations in clinical efficacy or safety [2, 3]. Therefore, the manufacturer of the biosimilar product is required to conduct phase III randomized controlled trials (RCT) aiming to demonstrate that those changes do not adversely affect the identity, purity,or potency of the potentially approved biologic product [4]. Notwithstanding, most health organizations do not consider biosimilar to be interchangeable with innovator product and recommend against substituting innovator and biosimilar agents in clinical practice.

FSH has served for decades as the active component in different pharmaceutical preparations for treatment of infertility, e.g., to induce ovulation in oligo-anovulatory patients or to stimulate the development and maturation of a large number of follicles in patients undergoing controlled ovarian hyperstimulation (COH) for in vitro fertilization (IVF). While urinary FSH (uFSH) was used as the active material of the early pharmaceutical preparations used, recombinant FSH (rFSH) preparations produced in Chinese hamster ovary (CHO) cells, or human cell lines have become available later on.

## FSH isoforms

It is well known that not only the levels of the FSH change during the different menstrual phases but also the composition of its different FSH isoforms. While Padmanabhan et al. [5] demonstrated that the rate of acidic isoforms in the serum is the lowest during the preovulatory and ovulatory phase; Ulloa-Aguirre et al. [6] found that the basic isoforms are secreted before ovulation. Zambrano et al. [7] have demonstrated that the proportion of the acidic isoform is higher during the early to mid-follicular phase compared to the preovulatory phase.

When FSH activity is measured in vitro, acidic isoforms of FSH have a lower activity than the more basic isoforms. In contrast, when the activity is measured in vivo, the acidic isoforms of FSH have a higher activity than the basic isoforms of the same preparation thus, reflecting higher affinity and efficacy of the basic isoforms, with shorter half-life in vivo.

## Measurement of FSH preparations activity

The activity of the FSH preparations can be measured in vitro or in vivo. Measurement of FSH activity in vitro can be performed by testing the efficiency of the binding of FSH to the membrane receptor, or by measuring the ability of FSH to stimulate enzymes or secondary messengers within target cells in culture. Measurement of the biological activity in vivo can be performed in animals and in humans. Since the 1950s, the most common and standard model for measuring the biological activity of FSH preparations in animals is the Steelman-Pohley assay, which is performed in two groups of immature female rats with low endogenous FSH level and is based on measuring ovary mass augmentation [8].

The activity of FSH preparations is influenced by the clearance rate of the hormone, which depends on the metabolism of different living organisms. Furthermore, in humans, exogenous FSH is distributed within the extracellular fluid space and the apparent volume of distribution and clearance are both proportional to body weight. Thus, body weight is inversely associated with follicular development and serum levels of E2 in response to FSH dosage. It is therefore not surprising that the activity, as determined in rats, will not be reliable in other organisms, as was clearly evident by De Leeuw et al. [9]. While comparing the biological availability and the half-life of rFSH and uFSH, they could not observed any differences between these two preparations, after calibration of an identical amount of International Units using the Steelman-Pohley assay, when they were injected into rats. However, a difference was found when injected into dogs [9]. They therefore concluded that the Steelman-Pohley assay is not a suitable model for predicting the biological activity of FSH in animals other than rats.

The fact that two FSH preparations contain the same number of activity units according to the Steelman-Pohley assay does not indicate anything regarding the number of moles of FSH in each of the preparations. Comparison of the efficacy of two preparations should be performed on a molar basis. For example, already in 2000, the European Health Authorities approved a change of the initial recommended dosage of the Puregon® from 75 IU to 50 IU. The reason for the amendment was that, the actual dosing advice was based on the dosages used for urinary FSH. Even though the two preparations had the same IU it was found that the Puregon® is more efficacious than urinary FSH and therefore may require a lower dose [10]. Moreover, administration of identical bioactive doses (based on the Steelman-Pohley in vivo rat bioassay) of rhFSH from a cell line of human fetal retinal origin and follitropin α resulted in slower clearance and thus, significantly higher follicular and endocrine responses as well [11]. A difference in the clearance of between rats and humans was believed to be the most likely explanation for the limited prediction of the in vivo rat bioassay for its potency in humans. Therefore, this novel recombinant product was dosed in microgram (μg) of protein content rather than in IU of biological activity.

These examples illustrate that the time has come that the measurement of the biological activity of FSH in humans should be required by other methods instead of the Steelman-Pohley assay. Specifically we believe that the determination of dose- response curves in well characterized populations of women for well-defined outcomes during COH in preparation for ART is needed

## Controlled ovarian hyperstimulation

The challenge with which infertility specialists are continuously encountering is tailoring patient’s treatment, e.g., the need for increased ovarian stimulation and recruitment of follicles on the one hand, while preventing the ovarian hyperstimulation syndrome (OHSS), on the other hand. “Tailoring” the treatment is based mainly on determining the initial daily FSH dose, which is based on several parameters, including patient’s age, body mass index (BMI), the etiology of infertility and measures of ovarian reserve [Day 3 serum FSH, antral follicle count (AFC) and/or random serum anti-mullerian hormone(AMH) level]. Furthermore, the daily FSH dose is adjusted according to the patient’s response to COH, as reflected by monitoring serum E2 levels and the number and size of the follicles recruited [12].

## FSH preparations

Regardless of the source of FSH, the protein structure is identical, while the glycosylation patterns, which result from differences in posttranslational modifications, vary. When a FSH preparation is separated into two or more preparations, those preparations with a higher degree of sialylation, i.e., acidic preparations, will have a longer half-life and higher in vivo activity, as compared to the basic preparations with a lower degree of sialylation. Mulders et al. [13] have examined the in vivo activity of different isoforms of rFSH in rats, which were separated by isoelectric focusing (IEF). They found that acidic isoforms were 100 to 200 times more active than basic isoforms. Similarly, D’Antonio et al. [14] examined the clearance rate of acidic isoforms compared to basic isoforms originating from the same rFSH preparation using a rat model. The acidic isoforms were found to have a lower clearance rate, which supports their higher biological activity in vivo (as determined by the Steelman-Pohley assay). In the discussion, the authors cited other studies demonstrating that the sialic acid content determines the clearance rate, the higher the sialic acid content in the molecule, the lower the clearance rate, and the half-life and biological activity in vivo increase accordingly. Similarly, Chappel [15], in a publication on the use of gonadotropin isoforms in different pharmaceutical preparations, proposed using acidic isoforms at the beginning of the treatment, in order to recruit follicles, and later to change to basic isoforms in order to control the number of follicles and prevent OHSS. Moreover, when a large number of follicles are required, the use of acidic isoforms, with the longer half-life is recommended.

Many studies and multiple meta-analyses comparing different FSH preparations have yielded conflicting results for ovarian stimulation variables and pregnancy rate [16, 17]. When examining the different commercial FSH preparations, the isoforms present in uFSH are more acidic than those in rFSH [18]. Interestingly, based on the natural distribution of FSH isoforms during the follicular phase of the menstrual cycle, Gurgan et al. [19] have demonstrated favorable outcomes and improved efficacy, while using a sequential administration of uFSH followed by rFSH compared with either FSH preparation alone. Moreover, when prospectively evaluating the efficacy of a protocol that mimics the physiological shift form an acidic to a less acid FSH isoform during oocyte maturation, Gerli and Di Renzo [20] could demonstrate that the combined protocol (hFSH + rFSH) resulted in significantly less IU of FSH necessary for ovarian stimulation together with shorter stimulation days and with higher number of oocyte yield, embryo quality, implantation and pregnancy rates.

## Biosimilar FSH preparations

Gonal-f™ (Follitropin alpha, Merck Serono S.A.) is the FSH innovator products that all the recently biosimilar products were referred and compared to. When Gonal-f™ was compared to a potential biosimilar, Grass et al. could demonstrate that the two r-hFSH preparations have apparently identical polypeptide chains but a somewhat different glycosylation pattern [21]. In particular, for the biosimilar, the N-terminal glycosylation site of the β-chain contained a higher percentage of tri- and tetra-antennary glycans and of N-acetyllactosamine repeats compared with Gonal-f™. A discrimination that may matter as under sialylation and increased numbers of antennae have an opposite effect on the biospecific activity of r-hFSH [22], with the consequent relevance for its biological activity.

The aforementioned different isoforms composition dictates to the manufacturer of the biologic product to conduct a complex and comparability studies, aiming to demonstrate that those changes do not adversely affect the purity, potency or the identity of the product. Following those studies the products may be introduced to clinical practice and the most crucial issue is whether they are identical to the innovator product or just another product. Whether they are “identical twins or siblings” will lead their being interchangeable, or not, with the innovator products.

## RCT comparing Gonal-f™ to a biosimilar products

Bemfola (follitropin alfa) (Finox AG, Switzerland), a new recombinant FSH, was recently introduced to clinical practice following an assessor-blinded, randomized, parallel group, multi-center, phase 3 trial aimed to test equivalence in the number of retrieved oocytes, with a power of 90 %, an alpha error of 2.5 % and a pre-determined clinical equivalence margin of ±2.9 oocytes for the relevant population [23]. Normal responders, young, good prognosis patients underwent the long GnRH agonist suppressive protocol with a daily FSH dose of 150 IU. Compared with Gonal-f, Bemfola treatment resulted in a statistically equivalent number of retrieved oocytes. Furthermore, other (but not primary end-points) observations were the similar clinical pregnancy rate per embryo transfer in first and second cycles with no difference in severe OHSS between treatment groups. The authors concluded that Bemfola can be an appropriate alternative in ovarian stimulation protocols.

A closer look at this RCT [23] (Table 1) reveals that while receiving the same daily and total rFSH doses, the Gonal-f group achieved non significantly more oocytes (10.7 vs 10.6, respectively), despite lower peak E2 levels and lower cancellation rate (7704 vs 8982pmol/L and 0.8 % vs 2.0 %, respectively) with the consequent decreased incidence of OHSS (3.3 % vs 5.6 %, respectively). Moreover, while clinical and ongoing pregnancy rates were non-significantly higher in the Gonal-f group (44.7 vs 36.1 and 41.5 vs 33.7, respectively), power analyses calculation reveals 44.6 % and 43.2 %, respectively, for the present sample sizes and an α − error of 5 %. Just emphasizing, that this study was underpowered and was not designed to refer to pregnancy rates. Of note, patients receiving Bemfola had a significantly higher chemical pregnancy rate, that did not develop to clinical pregnancy (10.4 vs 4.1 %, *p* < 0.02, respectively).

**Table 1:** Data from the Bemfola and Ovaleap RCTs [23–24)

	Bemfola [23]		GF [23]		GF [24]		Ovaleap [24]	
	# of patients	% of patients treated	# of patients	% of patients treated	# of patients	% of patients treated	# of patients	% of patients treated
# of patients	249		123		146		153	
BMI (kg/m^2^)	22.7		22.4		22.6		22.8	
FSH (IU)	6.9		6.9		7.3		7	
Duration of stimulation (days)	10.6		10.7		9.7		9.3	
Total dose of FSH used	1555		1569		1614		1536	
E2 (pmol/L)	8982		7704		9534		10070	
OHSS	14	0.056	4	0.032	4	0.027	7	0.045
# oocyte	10.7		10.4		12.1		12.2	
#ET	1.5		1.6		NA		NA	
FR (%)	66.1		64		NA		NA	
IR (%)	31.8		36.7		NA		NA	
Biochemical pregnancy	116	0.465	60	0.487	60	0.410	58	0.379
Clinical pregnancy	90	0.361	55	0.447	52	0.356	43	0.281
Ongoing pregnancy	84	0.337	51	0.414	49	0.335	42	0.274
LBR	NA		NA		47	0.321	41	0.267
Chemical pregnancy	26	0.104	5	0.040	8	0.054	15	0.098

Ovaleap® (follitropin alfa) is another new r-hFSH manufactured in CHO cells that has been developed as a biosimilar to Gonal-f. It was introduced to clinical practice following a multinational, randomized, active-controlled, assessor-blinded, parallel group patient study [24], aimed to test for the number of oocytes retrieved, as the primary efficacy endpoint, with a 90 % power and a two-sided level of α = 0.05. As with the previous Bemfola study, normal responders, young, good prognosis patients underwent the long GnRH agonist suppressive protocol with a daily FSH dose of 150 IU. The mean number of oocytes retrieved was equivalent between the two groups with comparable safety profiles.

Here again, a closer look at this RCT [24] (Table 1) reveals that while receiving the same daily and total rFSH doses, the Gonal-f group achieved non significantly lower peak E2 levels and lower cancellation rate (9534 vs 10070pmol/L, respectively) with the consequent decreased incidence of OHSS (2.7 % vs 4.5 %, respectively). Moreover, clinical, ongoing pregnancy and live birth rates were higher in the Gonal-f group (Table 1), however, non-statistically significant, due to the insufficient sample size.

Gathering the data from the aforementioned studies [23, 24] (Table 2), comparing Gonal f vs the biosimilar products, revealed non-significant 4 % difference in clinical and ongoing pregnancy rates, in favor of Gonal f over the biosimilar products, with half the incidence of OHSS (2.9 vs 5.2 %, respectively).

**Table 2:** Gathered data from the Bemfola and Ovaleap RCTs [23 + 24]

	Biosimilars [23 + 24]		GF[23 + 24]	
	# of patients	% of patients treated	# of patients	% of patients treated
# of patients	402		269	
OHSS	21	0.052	8	0.029
Biochemical pregnancy	166	0.412	120	0.446
Clinical pregnancy	142	0.353	107	0.397
Ongoing pregnancy	133	0.330	100	0.371

## Conclusions

The aforementioned figures were obtained while treating the “ideal” patients, young, good responder with the best prognosis. Therefore, further comparative studies are needed in other patient populations that are encountered during routine daily clinical practice, e.g., older, poor responders, patients with repeated IVF failures or high responders, such as those with polycystic ovary syndrome, before the universal implementation of biosimilar products to clinical use. Biosimilars are not exact copies, and are not considered generic versions of the reference product. They are actually a *regulatory synonym*, facilitating a fast track introduction of a FSH preparation to the COH armamentarium. We believe the time has come that the measurement of the biological activity of FSH in humans should require other methods than the Steelman-Pohley assay, such as the determination of dose–response curves for well characterized patient populations for well-defined outcomes during COH in preparation for ART. We therefore recommend against interchanging or substituting innovator and biosimilar agents in clinical practice, and believe that the decision whether to use an innovator or a biosimilar product, should be reserved to the discretion of the treating physician. This recommendation is in line with the recently published Australian Public Assessment Report [25], instructing the sponsor of a biosimilar product to add a ‘Dear Healthcare Professional Letter’. A letter that “should at least contain.... A statement that although Bemfola is considered biosimilar it is not interchangeable with other follitropin alfa products on an individual patient basis”.

## Abbreviations

AFC, antral follicle count; CHO, chinese hamster ovary; COH, controlled ovarian hyperstimulation; FSH, follicle stimulating hormone; GnTH, gonadotropin releasing hormonr; IVF, in vitro fertilization; OHSS, ovarian hyperstimulation syndrome; RCT, randomized controlled trial
