# Erratum: Biosimilar FSH preparations- are they identical twins or just siblings?

**DOI:** 10.1186/s12958-016-0194-5

**Published:** 2016-09-15

**Authors:** Raoul Orvieto, David B. Seifer

**Affiliations:** 1 Department of Obstetrics and Gynecology, Infertility and IVF Unit, Chaim Sheba Medical Center (Tel Hashomer), Ramat Gan, 52621 Israel; 2 Sackler Faculty of Medicine, Tel Aviv University, Tel Aviv, Israel; 3 Department of Obstetrics and Gynecology, Division of Reproductive Endocrinology and Infertility, Dartmouth-Hitchcock Medical Center, Geisel School of Medicine at Dartmouth, Lebanon, NH USA

## Erratum

The original version of this article [1] unfortunately contained an error.

The following lines regarding RCT [23] in Table 1: ‘…the Gonal-f group achieved non significantly more oocytes of (10.7 vs 10.6, respectively), despite lower peak E2 levels and lower cancellation rate (7704 vs 8982 pmol/L and 0.8 % vs 2.0 %, respectively) with…’ has been corrected.

This has been corrected to: ‘...the Gonal-f group achieved non significantly less oocytes (10.4 vs 10.7, respectively), with lower peak E2 levels and lower cancellation rate (7704 vs 8982 pmol/L and 8.9 % vs 11.2 %, respectively) with the consequent decreased incidence of OHSS…".
